# The effect of service outsourcing on labor income share: Measuring labor income share from the global value chains perspective

**DOI:** 10.1371/journal.pone.0309656

**Published:** 2024-09-11

**Authors:** Qi Ye, Jie Yan

**Affiliations:** 1 School of International Business, Southwestern University of Finance and Economics, Chengdu, China; 2 School of Economics and Management, Tsinghua University, Beijing, China; Istinye University: Istinye Universitesi, TÜRKIYE

## Abstract

Inspired by the positive impact of service outsourcing in Chery and other enterprises on human resources, this paper explores the impact of service outsourcing on labor income share. This paper introduces a framework to analyze how value added is distributed between capital and labor along the mix of inputs from different countries and sectors participating in global value chains and examines the effect of service outsourcing on the labor share income. Using the World Input-Output Database (WIOD) and OECD Inter-Country Input-Output (OECD- ICIO) table, this paper utilizes the WWZ decomposition method of global value chains (GVCs) to quantify labor share income. The results show that: (1) service outsourcing significantly contributes to the increase in labor share income; (2) Offshore outsourcing had a statistically stronger effect on labor share income after the financial crisis, both compared to the past and to onshore outsourcing; (3) Offshore outsourcing has a higher coefficient in countries with low technology. For ease of comparison, only onshore outsourcing shows a statistically significant difference among various service types; (4) The analysis using Chinese data reveals that the coefficient of offshore outsourcing is negative and statistically significant, indicating that industries with higher levels of service outsourcing have a lower labor share income.

## 1. Introduction

The OECD’s Balance Sheet spanning 1995 to 2019 indicates a gradual rise in countries engaging in service outsourcing collaboration. Chery Automobile has been making substantial contributions to the automotive industry, notably in China, achieving annual sales surpassing 1.88 million units and consistently ranking as the leading Chinese brand car exporter for 21 consecutive years. Chery has effectively reduced human resource management costs and tackled labor shortages through diverse outsourcing initiatives. For instance, Chery has outsourced R&D project development, technical personnel, and procurement tasks have been outsourced to multiple engineering tiers, supporting both vehicle and component development. Moreover, employee training has been outsourced to AVL. This collaborative model enhances research and development efficiency, reduces costs, and leverages specialized technical skills and resource advantages to foster innovation [1]. Human resource outsourcing at Chery has not only reduced its human resource costs but also driven innovation and boosted its operating profits, resulting in changes to its human capital structure. Outsourcing of human resource and consulting services has enhanced employee skills, thereby enhancing the company’s innovation and revenue-generating capabilities. The case of Chery Automotive exemplifies the recent trend in the evolution of service outsourcing content and the increase in labor income returns for enterprises. Understanding the impact of service outsourcing on labor income shares will shed further light on this evolving trend.

Since the early 1980s, a decline in the labor share income has been observed in the vast majority of countries and industries [2]. The labor share was measured for 94 countries and found that the global average labor share decreased from 0.70 in 1970 to 0.54 in 2009 [2]. The dramatic decline in labor share in China stands out among most countries [3, 4].

Global value chains (GVCs) denote the global production system wherein various stages of production are dispersed across different countries [5]. The motivation and significance of participating in GVCs for a country are multifaceted. They not only foster economic growth but also afford opportunities for countries to specialize in production stages where they possess a comparative advantage. China holds a pivotal position in GVCs owing to its expansive manufacturing sector and integration into global markets. A comprehensive understanding of China’s role in GVCs is essential for grasping the intricacies of the global economy.

Porter [6] originally proposed the term "value chain" to describe the sequence of activities undertaken by companies, from product design to consumer use. Thus, the concept of GVCs suggests that these production steps occur across multiple countries [7], heralding a new era of globally manufactured products. Extending from Solow’s foundational research [8], a substantial portion of the literature has concentrated on the intra-industry and sectoral disparities in labor income distribution [9]. In terms of both Gross Domestic Product (GDP) and international trade volume, China and the US rank the top two in the world. However, they are in completely different GVCs positions [10]. China, as one of the prominent participants in GVCs, represents an intriguing case study. The asymmetric interaction between manufacturing and service sectors in the two countries is caused by the functional differences between China and the US in international specialization, which ultimately leads to the differentiation of the GVC positions of the two countries [11]. The distance between China and the US in the GVCs is gradually widening, Dai et al. [11] explained the difference between China and the US from the perspective of industrial structure, but few literatures studied it from the perspective of factor income share.

The significance of this paper is to deconstruct the causes of the decline in labor income share from GVCs’ perspective. Service outsourcing plays a critical role in the value chain, influencing labor income distribution. Understanding the impact of service outsourcing on labor income share is essential. It helps in addressing structural imbalances in national income distribution and promoting balanced social development. Building upon existing models and methods, this paper constructs a method to measure labor share income from gross exports. It addresses a research gap in two distinct areas. First, the labor income share from domestic value added (DVA) is measured in this paper. This paper also looks into how labor income share is affected by service outsourcing.

The remainder of the paper is organized as follows. *The Literature review* summarizes the related literature and points out the research gap. The *Research design* section elaborates the decomposition of the gain from GVCs and shows the empirical model and information of the variables. The *Empirical Analysis* section describes the empirical model and the specification strategy, and empirical results are illustrated in the *Empirical Results* subsection. The *Further Analysis in China* section conducts the estimation using data from China to further study the effect of service outsourcing in a specific country. The *Conclusion* section concludes the paper.

## 2. Literature review

At present, there is little research on service outsourcing and labor income share. In this paper, considering the value-added decomposition method to measure the share of factor income and its relationship with service outsourcing, the related literature is reviewed.

### 2.1 GVCs decomposing

Johnson and Noguera [12] described value-added exports as the destination where the value added produced in each source country is absorbed. Los et al. [13] decomposed gross exports into three components, namely DVA, foreign value added, and re-exported value added, based on the concept of value added in exports. Koopman, Wang, and Wei [14], henceforth referred to as KWW, significantly advanced this area by providing a unified framework to decompose a country’s gross exports into nine value-added and double-counted components. Building on the KWW method, Wang et al. [15] expanded their decomposition method, proposing a multilateral approach applicable to various levels, including country/sector, bilateral, and bilateral/sector. This approach established a comprehensive set of accounting rules from official trade value statistics to trade value-added statistics, based on value addition, forming a complete national economic accounting system.

The decomposition result of the WWZ method for gross exports is the most detailed among existing methods [16–18]. This level of detail is crucial for accurately decomposing factor income share. Therefore, this paper outlines a general methodology that encompasses the measures introduced by Johnson and Noguera [12] (value added consumed abroad) and Los et al. [13] (value added in exports). This paper decomposes factor income in the value-added framework of the WWZ method.

A growing number of scholars used the added value approach to investigate the issues of labor market [9, 19, 20]. Despite extensive research on the subject, there is limited consensus in the literature regarding factor income from a GVCs perspective. Hummels et al. [21] stressed the significance of comprehending the role of international trade in the growth of services. Their findings underscored the growing importance of service sectors in global trade and the necessity to integrate these dynamics into value-added decomposition frameworks. Additionally, Antràs and Helpman [22] introduced the concept of “fragmentation” in international trade, stressing the significance of outsourcing and offshoring in the disaggregation of value added across borders. These studies collectively emphasize the necessity for robust methodologies to precisely quantify the value added within GVCs and their implications for global trade dynamics.

### 2.2 Service outsourcing and labor income share

Amid rising global competitive pressure, companies are encouraged to focus on core competencies and leverage outsourcing to benefit from others’ expertise [23]. Studies, exemplified by those showing that offshore outsourcing enhances production fragmentation and firm productivity while expanding the global distribution of goods and services through resource reallocation in GVCs [9–11].

This study is linked to research that examines factors influencing the labor market and labor income distribution. A growing body of research on factor income shares is driven by the degree of competition [24], changes in technology [25], foreign direct investment (FDI) flows [26], industry concentration [27, 28], capitalization of intellectual property product [29], relative price of investment [25], tax policy [30, 31], and non-market forces are the main causes [32, 33].

A considerable amount of empirical research was dedicated to examining the direct impact of offshore outsourcing on labor market outcomes [34, 35]. Additionally, offshoring was found to enhance firms’ productivity, enabling them to expand their workforce [36, 37]. According to studies on the impact of services outsourcing on the labor market, outsourcing exacerbated the income gap by increasing the earnings of high-skilled workers while reducing those of unskilled workers [38–40]. In developed economies, outsourcing predominantly impacts the income distribution between unskilled and high-skilled labor, while in emerging economies, it primarily affects the distribution between labor and capital [41]. Discussions on the effects of offshore outsourcing often center around labor markets due to their links to changes in employment, wage levels, and relative wages [21]. According to Amiti and Wei [42], the increase in offshore outsourcing has led to a decrease in labor income share in the United States, especially in industries with rapid outsourcing growth. This underscores the substantial impact of outsourcing on shaping income distribution patterns within economies. Slaughter [43] offered insights into the labor market impacts of international trade, illustrating that outsourcing, especially in manufacturing, resulted in job displacement and wage reduction for less-skilled workers. Nevertheless, existing research has not yet achieved a comprehensive understanding of the response of labor income share to services outsourcing.

This paper offers distinct contributions to the literature on service outsourcing and labor income share by presenting a refined analytical framework that delineates the distribution of value added within GVCs. Unlike prior studies, our approach, grounded in the WWZ decomposition method, affords a granular assessment of labor share income, enhancing the precision of our empirical inquiries. Our empirical findings underscore the robust impact of service outsourcing on labor income share, with a pronounced effect for offshore outsourcing post-financial crisis, particularly in nations with nascent technological sectors. This temporal and technological dimension provides fresh insights, distinguishing our work from existing analyses. Additionally, we address a significant research gap by quantifying labor income share from DVA, a largely unexplored area, thereby enriching the discourse on the subject. The inclusion of a case study on China, a global economic powerhouse, further distinguishes our research. It provides a nuanced perspective on the varied impacts of service outsourcing, acknowledging the heterogeneity across different economic landscapes. In sum, our paper advances scholarly understanding by dissecting the intricate mechanisms linking service outsourcing to labor income share, examining differential effects, and proposing specific policy implications in China.

## 3. Research design

### 3.1 Construction of the labor income share

**Fig 1:**
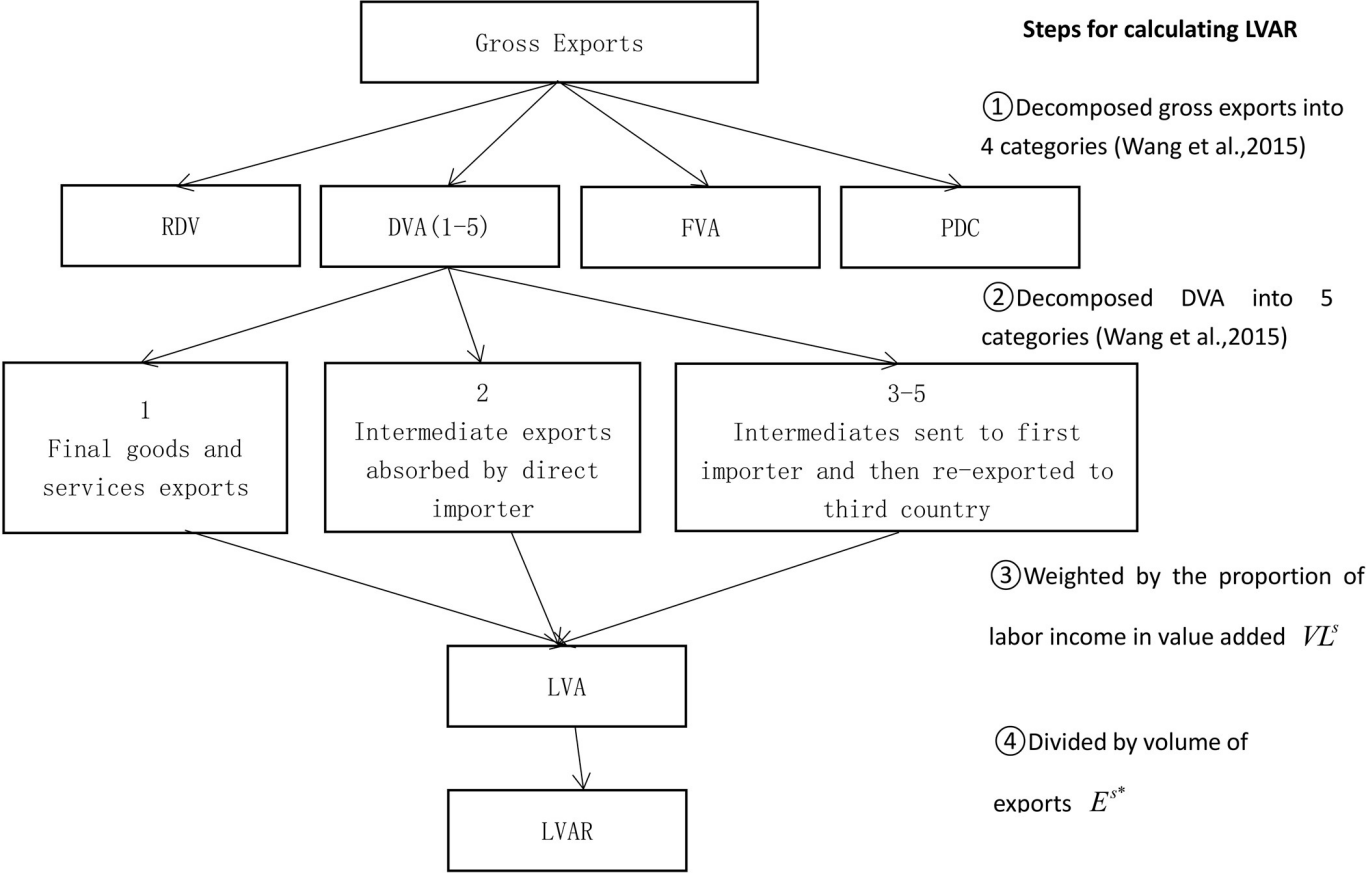
Flowchart of measuring labor income share.

Building upon the definition provided by Wang et al. [15], this paper presents a framework for decomposing the value added ratio from gross exports, allowing for the tracing of the sources and destinations of labor income across economies. Fig 1 depicts the incorporating flowchart. This decomposed indicator enables the assessment of the value added of each economy in terms of labor income share. This methodology is widely employed in the literature focusing on the benefits of value chain upgrading using global Input-Output tables [44].

This paper begins by investigating how labor income unfolds along GVCs through the construction of a matrix of the value added generated by labor. This matrix enables the decomposition of the value added into two portions, corresponding to labor and capital. It is assumed that there are *G* economies in the world (*s*, *r*, *t* = 1, 2, ⋯, *G*), and each economy has *N* industrial sectors (*i*, *j* = 1, 2, ⋯, *N*). *DVA* included in gross exports of the country *s* is denoted as

DVAs*=∑r≠sGVsBss1T#Ysr+VsLss2T#AsrBrrYrr+VsLss3T#Asr∑t≠s,rGBrtYtt+VsLss4T#AsrBrr∑t≠s,rGYtt+VsLss5T#Asr∑t≠s,rG∑u≠s,tGBrtYtu. (1)

The matrix $DVA^{s*}$ of cross-economies is derived by the product of coefficients *V*^*s*^ by the total value added. Where *V*^*s*^ captures the value added component *VA*^*s*^ derived from the labor of the country *s* as a proportion of gross output; the matrix *Y*^*tu*^ represents domestic final demand flowing from economy *u* to economy *t*; the matrix *A*^*sr*^ represents the input-output coefficient of domestic region *r’s* demand for intermediate goods in region *s*; *B* is the Leontief Inverse matrix; the notation # indicates that the corresponding elements of the matrix are multiplied together.

The vector *VL*^*s*^ allows solving the proportion of labor income in value added in each economic sector of the county *s*. Then, the labor value added (*LVA*) included in *DVA* can be obtained according to the matrix above.


LVAs*=∑r≠sGVLs#VsBss1T#Ysr+VLs#VsLss2T#AsrBrrYrr+VLs#VsLss3T#Asr∑t≠s,rGBrtYtt+VLs#VsLss4T#AsrBrr∑t≠s,rGYtt+VLs#VsLss5T#Asr∑t≠s,rG∑u≠s,tGBrtYtu. (2)

Given $E^{s*}$, the total export volume of the county *s*, the labor share income decomposed from gross exports can be denoted as follows

$$\begin{array}{c} LVAR^{s}=\frac{LVA^{s*}}{E^{s*}} \end{array}.$$ (3)

Building upon the KWW approach, this paper distinguishes two main components of value added. *DVA* can be divided into component derived from labor, denoted as *LVAR*, and component derived from capital, denoted as *CVAR*.


$$\begin{array}{c} DVAR^{s}=CVAR^{s}+LVAR^{s} \end{array}.$$ (4)

**Fig 2:**
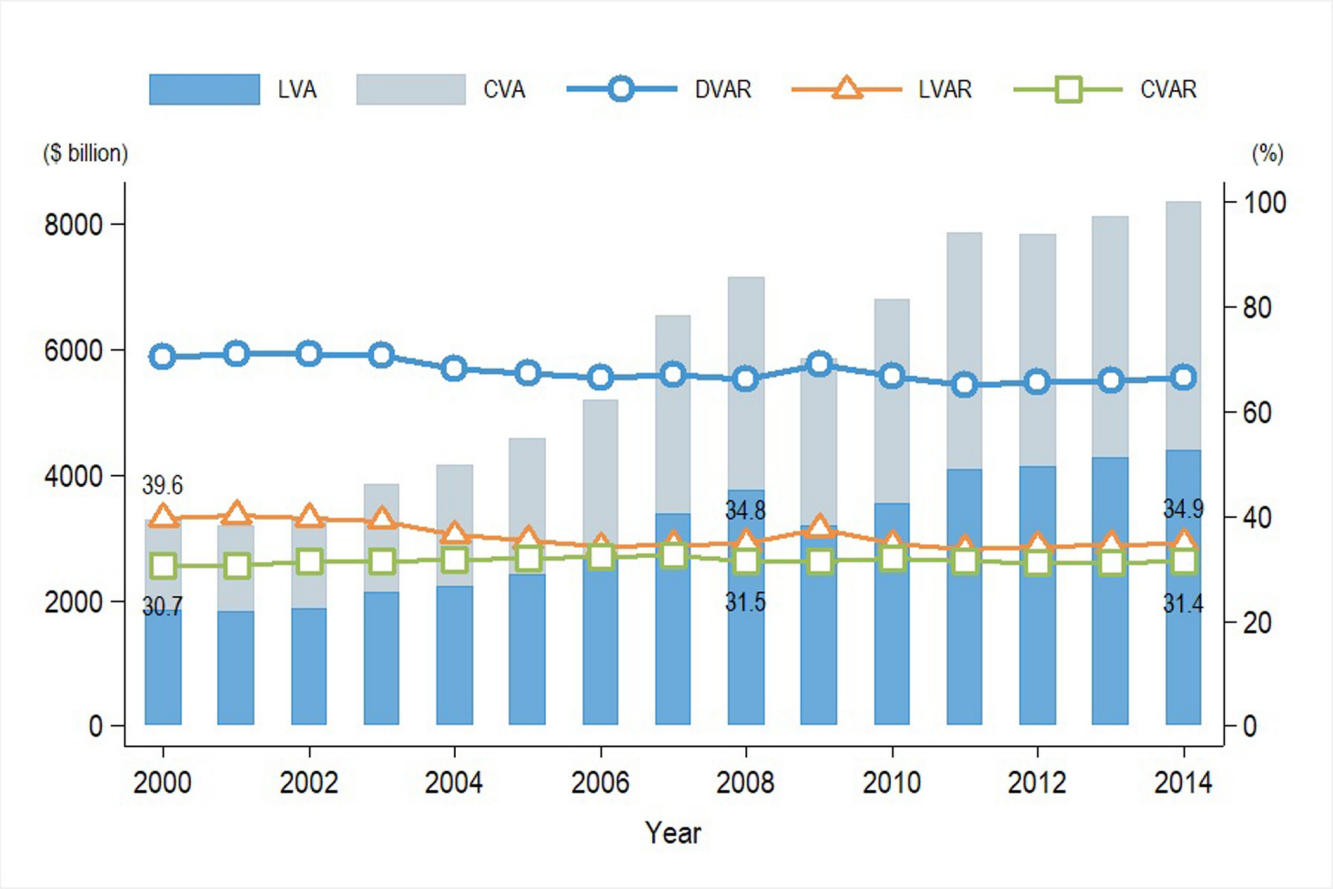
Composition of DVA in global gross exports in manufacturing industry: 2000–2014. Date Source: the latest version of the WIOD and Socio-Economic Accounts.

This paper presents an international comparison of the composition of value added in exports. Fig 2 illustrates the trends in the composition of DVA in global manufacturing exports from 2000 to 2014. First, on average, the labor share income is higher than the capital income share globally. Secondly, the global manufacturing *LVA* was declining, decreased from 39.6% to 34.9% over the period.

**Fig 3:**
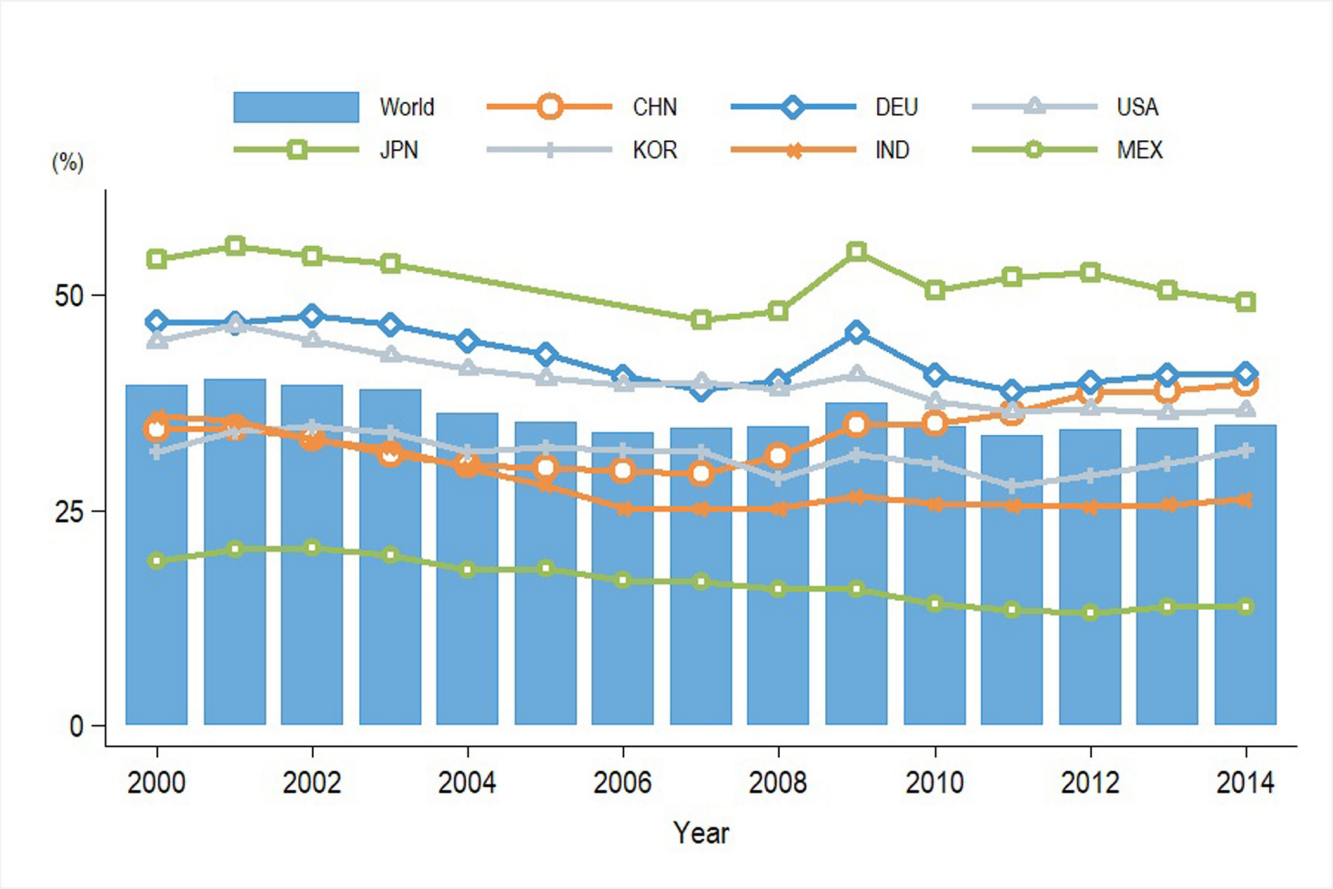
Labor income share decomposed from gross exports in manufacturing industry of major countries: 2000–2014. Date Source: the latest version of the WIOD and Socio-Economic Accounts. Measured by the authors.

Fig 3 illustrates the trend of variation in *LVAR* across major manufacturing industries from 2000 to 2014. The manufacturing *LVAR* of countries such as the United States, Germany, Japan, and others aligned with the average global level change, reflecting the overarching trend of increasingly deepening global production division under the lens of economic globalization [45].

Additionally, China’s manufacturing sector witnessed a 5.26% decrease in LVAR, falling from 34.51% to 29.25%, during the period from 2000 to 2007. It is noteworthy that China’s manufacturing LVAR remained below the global average and exhibited a downward trend until 2008, after which it began to increase. Importing foreign intermediate goods did not elevate the share of labor income in the domestic market, indicating a "squeeze effect" subsequent to China’s accession to the World Trade Organization (WTO). Domestic labor might have been displaced by imported foreign intermediate goods, thereby negatively affecting the distribution of labor income.

### 3.2 Empirical model

To examine the effect of service outsourcing on the value-added from the labor perspective, a cross-country panel regression model at the country-industry level is constructed as follows.

$$\begin{array}{c} LVAR_{ijt}=\beta_{0}+\beta_{1}O_{ijt}+\beta_{2}Controls+D_{i}+D_{j}+D_{t}+\varepsilon_{ijt}. \end{array}$$ (5)

where the subscripts *i*, *j* and *t* denote the country, industrial sector and year, respectively. *LVAR*_*ijt*_ refers to the share of value added derived from labor in exports. *O*_*ijt*_ refers to the level of service outsourcing, and is divided into *Offshore*_*ijt*_ and *Onshore*_*ijt*_ according to different classification of service outsourcing. *Controls* refers to a set of control variables, including characteristics in country level and industry level. The specification also controls for country fixed effects *D*_*i*_, industry fixed effects *D*_*j*_, and time fixed effects *D*_*t*_. The coefficient *β*_1_ identifies the average effect of service outsourcing on the share of value added derived from labor.

### 3.3 Data and settings of the indicators

This subsection concentrates on defining variables, measuring service outsourcing, and identifying control variables in the empirical part. The primary data utilized in this study are sourced from the following datasets.

#### OECD Inter-Country Input-Output (OECD-ICIO) tables

OECD-ICIO database used in this paper is the latest dataset provided by the OECD. The 2021 edition of OECD-ICIO tables has 45 unique industries based on ISIC Revision 4. Tables are provided for 66 countries for the years 1995 to 2018.

#### WIOD

The most recent version of the publicly released 2016 WIOD dataset encompasses 43 economies, comprising 15 major economies and 28 EU member economies, along with 56 industrial sectors. The labor share is sourced from the companion socioeconomic accounts database released by WIOD.

#### World Integrated Trade Solution (WITS)

The actual effective tariff, based on bilateral tariff rates, is utilized from 1988 to 2022 in the WITS database tariff rates [46]. ISIC Rev3 industries are matched with ISIC Rev4 industries, followed by the calculation of import and export tariff rates at the 2-digit ISIC industry level for OECD-ICIO countries (regions), weighted by corresponding import amounts.

#### World Development Indicators (WDI) database

The WDI database, provided by the World Bank, offers statistics on key development indicators, encompassing economic policies and financial aspects, for 266 countries and regions spanning from 1960 to 2020.

Many studies have heavily relied on world input-output tables to construct indicators of value added embodied in intermediate trade flows, which are subsequently transformed into the required variables. Industry tariffs data are sourced from the WITS database. Data on trade openness (*Exim*), foreign direct investment (*Fdii* and *Fdio*), physical capital investment (*Ftot*), and information infrastructure (*Pnet*) are obtained from the WDI database.

A brief introduction on the measurement of the indicators as follows.

#### Labor income share

This paper decomposes labor income share in value added from gross exports. The specific decomposition is described in the previous section.

#### Service outsourcing

The existing literature on service outsourcing classification continues to categorize it into multiple types. Most of the studies categorized service outsourcing into "Onshore Outsourcing" and "Offshore Outsourcing" [40, 47]. Feenstra and Hanson [44] categorized it as "Domestic Outsourcing" (onshore) and "Offshore Outsourcing". This paper contributes to the discussion by examining the distinction between "Onshore" and "Offshore" outsourcing and analyzing their effects on labor income share.

In this paper, the OECD-ICIO table is used to measure service outsourcing. First, the offshoring service outsourcing indicator is constructed [44], as follows.

$$\begin{array}{c} Offshore_{i}=\sum_{j}\left(\frac{X_{ij}}{Y_{i}}\times \frac{M_{j}}{D_{j}}\right) \end{array}$$ (6)

where *X*_*ij*_ is the fraction of intermediate service inputs from the service industry that are used in manufacturing production. *E*_*j*_ represents the total amount of all factor inputs used in manufacturing production. *M*_*j*_ and *D*_*j*_ refer to total imports and total consumption in the service industry, respectively. $D_{j}=P_{j}+M_{j}-E_{j}$, where *P*_*j*_ represents the total output of the service industry, *M*_*j*_ is the total import of the service industry, and *E*_*j*_ refers to the total export of the service industry.

The onshore service outsourcing indicator is constructed as follows. Noted that the onshore outsourcing portion is the remaining portion excluding the import portion. Therefore, onshore service outsourcing is expressed as

$$\begin{array}{c} Onshore_{i}=\sum_{j}\left(\frac{X_{ij}}{Y_{i}}\times \left(\text{1-}\frac{M_{j}}{D_{j}}\right)\right). \end{array}$$ (7)

The control variables introduced in this paper include industry size (*Output*), industry tariffs (*Traiff_ext* and *Traiff_imp*), trade openness (*Exim*), foreign direct investment (*Fdii* and *Fdio*), physical capital investment (*Ftot*), and information infrastructure (*Pnet*). For saving place, the estimation results of control variables are uncovered at the result tables. The descriptive statistics and definitions are shown in Table 1.

**Table 1:** Statistical description of variables.

Type of variable	Variables	Obs	Mean	Std	Min	Max
Explained variable	*lvar*	10552	0.3714	0.1177	0.0386	0.9814
Explanatory variable	*Offshore*	10552	0.0391	0.0353	-0.0327	0.3530
	*Onshore*	10552	0.3230	0.0966	0.0471	0.7993
Control variable	*Output*	10552	8.7712	2.1921	0.0783	14.3168
	*Traiff_ext*	10552	0.0681	0.0474	0.0000	1.5381
	*Traiff_imp*	10077	0.0411	0.0603	0.0000	0.7223
	*Exim*	10552	0.4563	0.2669	0.0910	1.5956
	*Fdii*	10520	0.0957	0.3422	-0.5761	4.4908
	*Fdio*	10520	0.0718	0.2808	-0.8723	3.0125
	*Ftot*	10552	0.2347	0.0496	0.1083	0.4452
	*Pnet*	10501	0.5175	0.2650	0.0053	0.9630

## 4. Empirical results

### 4.1 Benchmark results

The benchmark estimation results are presented in Table 2. The first five columns present Ordinary Least Squares (OLS) estimates, while the last two columns display two-stage least squares estimation (2SLS) results. This paper begins with a simple OLS specification that includes only industry, country fixed effects, and year fixed effects in columns 1 and 3. The coefficients of *Offshore* and *Onshore*, are both statistically significant and positive. The result indicates that both onshore and offshore service outsourcing can increase a country’s labor income share. Given that countries with higher levels of outsourcing of services experienced greater growth in labor income share.

**Table 2:** Benchmark results.

Dependent variable: LVAR	OLS					2SLS	
	(1)	(2)	(3)	(4)	(5)	(6)	(7)
*Offshore*	0.7589***	0.6526***			0.4645***	1.0000***	0.1536***
	(0.0939)	(0.0910)			(0.1041)	(0.1459)	(0.0166)
*Onshore*			0.2594***	0.1460***	0.1047***		
			(0.0177)	(0.0150)	(0.0172)		
*L*.*offshore*						0.5733*** (0.0415)	
*L*.*onshore*							0.8989*** (0.0072)
*p*-value of H_0_: ${\tilde{\beta }}_{off}$ = ${\tilde{\beta }}_{on}$ = 0					0.0016**		
						
Controls	No	Yes	No	Yes	Yes	Yes	Yes
Fixed effects	Yes	Yes	Yes	Yes	Yes	Yes	Yes
R square	0.6521	0.7060	0.6603	0.7060	0.7079		
Observations	10552	9994	10552	9994	9994	10552	9994

Robust standard errors are in parentheses. *p*-value are based on the F-test of the H_0_. ***, **, * stand for the significance level at 1%, 5% and 10%.

In columns 2 and 4, this paper introduces additional characteristics as control variables. Specifically, include both regressors of interest in the same equation and present the results in column 5. The impact of offshore service outsourcing is notably stronger than that of onshore service outsourcing. The stronger impact of offshore service outsourcing suggests that integrating with the global labor market can lead to more significant changes in labor income distribution. Managers should consider how their outsourcing strategies can leverage global labor market opportunities to enhance overall labor productivity and income. Thus far, one might be concerned that other issues could arise from OLS. Subsequently, this paper uses 2SLS by introducing lagged instruments for offshore service outsourcing and onshore service outsourcing (*L*.*offshore*, *L*.*onshore*). The instrumental variable estimation results are presented in columns 6 and 7. Clearly, the results are robust to these additional controls and approach.

### 4.2 Robustness checks

In this subsection, this paper presents a series of robustness checks on some econometric concerns. Regression results are reported in Table 3.

**Table 3:** Robustness checks.

Dependent variable:	*LPLV*		*LVAR*			
	(1)	(2)	(3)	(4)	(5)	(6)
*Offshore*	27.0339**		0.6343***		0.0343***	
	(13.1517)		(0.0926)		(0.0058)	
*Onshore*		2.4502***		0.1387***		0.0240***
		(0.7790)		(0.0155)		(0.0043)
Controls	Yes	Yes	Yes	Yes	Yes	Yes
Fixed effects	Yes	Yes	Yes	Yes	Yes	Yes
R Square	0.1110	0.1052	0.7056	0.7055	0.7032	0.7026
Observations	9993	9993	9994	9994	9994	9994

*Controls* includes all the above-mentioned control variables. *Fixed effects* include year fixed effects, country fixed effects, and industry fixed effects. ***, **, * stand for the significance level at 1%, 5% and 10%.

#### Alternative measure of outcome variable

—One concern is that relying solely on the share of labor income in exports may not sufficiently capture the influence of labor on the value chain component. To address this concern, this paper explores an alternative measure of the outcome variable: the forward-linkage labor production length of value added (*LPLV*). The estimated results are presented in columns 1 and 2 of Table 3. The coefficient of the regressor of interest consistently exhibits positive and statistically significant values, suggesting that the benchmark results are not influenced by any particular measure of the outcome variable.

#### Alternative measure of regressor of interest

—Another potential concern arises from the difference between the definitions of servicing outsourcing provided in the Manual on Statistics of International Trade in Services 2010 (MSITS, 2010) and the Balance of Payments Manual which is utilized in the benchmark results; notably, the latter excludes the construction industry. To assess whether this discrepancy in definition affects the accuracy of the results, this paper performs a robustness check using the definition of outsourcing of services from MSITS 2010. The estimation results, presented in columns 3 and 4 of Table 3, continue to reveal a positive and statistically significant effect of service outsourcing.

#### Alternative classification of service outsourcing

—To address concerns regarding aggregation bias, this paper approximates the magnitude of offshore service outsourcing (onshore service outsourcing) development by utilizing the combined value of other business services and imports (exports) of computer and information services. The regression results, presented in columns 5 and 6 of Table 3, are as follows. The benchmark results remain robust to the alternative classification of service outsourcing, alleviating concerns regarding any potential complications arising from this classification.

### 4.3 Mechanisms

The labor market typically serves as the primary channel influencing the distribution of labor income. Several studies indicate that outsourcing enhances the alignment between managers and employees, resulting in heightened employment levels [48]. Expanding upon this research, this paper investigates whether the labor market serves as the mechanism through which service outsourcing influences the distribution of value-added derived from labor. For brevity, this paper concentrates on the estimation specifications in Table 4 and solely presents first-stage results for the mechanisms (second-stage results are presented in **S2 Appendix**).

**Table 4:** Mechanism.

Dependent variable:	(1)	(2)	(3)	(4)
	*LnEMP*	*LnEMP*	*LnEMPE*	*LnEMPE*
*Offshore*	1.6271***		2.0283***	
	(0.5936)		(0.5847)	
*Onshore*		0.2165**		0.2656***
		(0.0912)		(0.0924)
Controls	Yes	Yes	Yes	Yes
Fixed effects	Yes	Yes	Yes	Yes
R Square	0.9468	0.9467	0.9391	0.9389
Observations	9994	9994	9994	9994
Dependent variable:	(5)	(6)	(7)	(8)
	*LnH_EMPE*	*LnH_EMPE*	*LnINCOME*	*LnINCOME*
*Offshore*	2.1952***		4.3314***	
	(0.6047)		(0.6262)	
*Onshore*		0.3144***		0.4507***
		(0.0934)		(0.0979)
Controls	Yes	Yes	Yes	Yes
Fixed effects	Yes	Yes	Yes	Yes
R Square	0.9446	0.9445	0.9831	0.9829
Observations	9994	9994	9994	9904

*Controls includes* all the above-mentioned control variables. *Fixed effects* include year fixed effects, country fixed effects, and industry fixed effects. Robust standard errors are in parentheses. ***, **, * stand for the significance level at 1%, 5% and 10%.

To reinforce the labor market’s role in driving the main results, this paper initially examines if the industrial labor market size, measured by the number of workers in the corresponding industry (*LnEMP*), expanded with the growth of service outsourcing. As depicted in columns 1 and 2 of Table 4, this finding aligns with Antràs et al. [48], suggesting that service outsourcing positively impacts the industrial labor market’s size. To formally examine this labor market size pathway, this paper introduces another variable, the number of employees (*LnEMPE*), as a measure of labor market size. As shown in columns 3 and 4, the results suggest that a one unit increase in the offshore service outsourcing leads to a 2.0283 percentage point increase in labor income share, which is statistically significant at the 1% level. Offshore service outsourcing demonstrates a more substantial and statistically significant impact on labor market size compared to onshore service outsourcing, mirroring the influence of service outsourcing on labor income share.

To reinforce the assertion that the labor market enhancement channel is instrumental in driving the impact of service outsourcing, this paper also uses the total hours worked by employees (*LnH_EMPE*) and presents the findings in columns 5 and 6. Since the dataset contains comprehensive labor information, this paper also employs employee income (*LnINCOME*) to ascertain if the benchmark results are influenced by labor income. In columns 7 and 8 of Table 4, the coefficients of service outsourcing persistently display positivity and statistical significance, which proves that service outsourcing affects the labor income share by adjusting the labor market. channel.

### 4.4 Heterogeneous effects

For a deeper comprehension of how service outsourcing enhances the labor income share across various economies, this paper analyzes the impact of service outsourcing separately in OECD and non-OECD countries. Upon comparing columns 1 and 2 with columns 3 and 4 in Table 5, this paper observes analogous outcomes for OECD and non-OECD countries, indicating no statistically significant disparity. When examining the impact of service outsourcing in non-OECD countries, a statistically significant distinction is found between the two forms of outsourcing, wherein offshore outsourcing exhibits a more pronounced effect on enhancing the labor income share. This finding implies that firms in both OECD and non-OECD countries should customize their services outsourcing strategies according to their level of economic development and labor market characteristics. For example, non-OECD countries could give more consideration to how to increase the share of labour income through offshoring.

**Table 5:** Heterogeneity.

Dependent variable: LVAR	OECD		Non-OECD	
	(1)	(2)	(3)	(4)
*Offshore*	0.3968***		0.8205***	
	(0.0846)		(0.1653)	
*Onshore*		0.1205***		0.1553***
		(0.0165)		(0.0399)
*p*-value for H_0_: *${\tilde{\beta }}_{\left(1\right)}$* = ${\tilde{\beta }}_{\left(2\right)}$	0.0100	0.1600		
*p*-value of H_0_: ${\tilde{\beta }}_{off}$ = ${\tilde{\beta }}_{on}$ = 0	0.8921		0.0013**	
Controls	Yes	Yes	Yes	Yes
Fixed effects	Yes	Yes	Yes	Yes
R Square	0.7668	0.7687	0.5614	0.5523
Observations	7759	7759	2235	2235

*Controls* includes all the above-mentioned control variables. *Fixed effects* include year fixed effects, country fixed effects, and industry fixed effects. ${\tilde{\beta }}_{\left(1\right)}$ refers to the coefficient of group 1, here means the group of OECD. *p*-value are based on the F-test of the H_0_. Robust standard errors are in parentheses. ***, **, * stand for the significance level at 1%, 5% and 10%.

To comprehend the divergent outcomes across various periods, industries, and service types, this paper examines the associated estimates. Taking the financial crisis in 2008 into account, offshore outsourcing exhibits a statistically stronger impact on the labor income share compared to previous periods before 2008 and onshore outsourcing. In nations with lower technological advancement, offshore outsourcing demonstrates a higher coefficient. Simplifying comparison, this paper notes that solely onshore outsourcing exhibits a statistically significant disparity among various service types. Detailed results are provided in the **S2 Appendix**.

In the analysis of heterogeneous effect, this paper finds that (1) offshore service outsourcing has a higher positive effect on the labor income share of non-OECD countries than onshore service outsourcing; (2) After the financial crisis in 2008, the positive effect of offshore service outsourcing on labor income share has improved, but it is less than that of onshore service outsourcing; (3) Service outsourcing in industries with higher technical level has a higher effect on increasing the share of labor income. Moreover, for low-tech industries, the offshore service outsourcing effect is higher than onshore service outsourcing; (4) Onshore business services outsourcing plays a stronger role in increasing the share of labor income than onshore non-business services.

To enhance the domestic labor market and share of labor income, this paper provides some suggestions according to the empirical results. Firstly, governments can foster the development of the service outsourcing industry through supportive measures like tax incentives and streamlined supervision. Enterprises should also be incentivized to expand their service outsourcing operations.

Secondly, countries with underdeveloped or low-tech industries should deepen collaboration with other nations, increasing the outsourcing of services abroad. Targeted support and incentives should be provided to domestic service outsourcing activities to elevate the quality of local enterprises engaging in outsourcing.

## 5. Further analysis in China

This paper analyses the global average effects of service outsourcing on the share of labor income by expanding the labor market size, total employee hours worked, and employee income. Nonetheless, divergent perspectives exist regarding whether service outsourcing results in domestic job losses [38, 49, 50], implying the necessity of distinct analyses for individual countries. Global Chinese manufacturing service outsourcing remained relatively limited, primarily concentrated in low-level service segments among manufacturing enterprises [51]. Considering the emphasis on diverse impacts among countries, conducting country-specific analyses becomes imperative.

According to China Service Outsourcing Development Report 2021 [52], Chinese enterprises engaged in offshore service outsourcing contracts valued at USD 171.68 billion (approximately RMB 1.1 trillion), marking a 22.3% year-on-year increase and constituting 53.3% of all service outsourcing contracts in 2021. The execution amount reached USD 130.31 billion, reflecting a 23.2% year-on-year increase and a 14.0% rise from the previous year, accounting for 57.5% of the total service outsourcing execution amount. Simultaneously, enhancing workers’ income and remuneration has consistently been a governmental priority [52]. During the October 2007 report of the 17th National Congress of the Communist Party of China, the proposition to "gradually increase the proportion of residents’ income in the national income distribution and enhance the proportion of labor remuneration in the initial distribution" was introduced. Subsequently, enhancing labor remuneration has emerged as a prominent theme in the reports of subsequent national congresses. Nevertheless, numerous studies indicated a downward trend in labor income share in China [3, 4]. Investigating the impact of service outsourcing on enhancing China’s labor income share can inform and enhance policy formulation.

This paper further investigates the impact of service outsourcing on the Chinese labor market and value-added structure by analyzing data from 2000 to 2014 across 17 manufacturing industries in China. This allows us to assess if the effects of service outsourcing in China differ from global patterns. The regression results are presented in Table 6. In column 1, the negative and statistically significant coefficient suggests that industries with higher offshore service outsourcing levels exhibit a reduced labor income share. This finding contrasts with observations from cross-country data. Importantly, the coefficient of onshore service outsourcing is statistically insignificant in the Chinese context.

**Table 6:** Further analysis.

Dependent variable:	LVAR	LVAR
	(1)	(2)
*Offshore*	-8.4339***	
	(1.6845)	
*Onshore*		-0.2681
		(0.1634)
Controls	Yes	Yes
Fixed effects	Yes	Yes
R Square	0.9157	0.9005
Observations	215	215

*Controls* includes all the above-mentioned control variables. *Fixed effects* include year fixed effects, country fixed effects, and industry fixed effects. Robust standard errors are in parentheses. ***, **, * stand for the significance level at 1%, 5% and 10%.

Several factors contribute to the disparity between the two results. First, China’s increased involvement in the international division of labor has reduced its labor income share. The expansion of offshore service outsourcing accelerates global production fragmentation, leading to a decrease in China’s labor income share. Second, despite being a large developing country with a high degree of export specialization, China’s level of specialization in outsourcing technical, marketing, and R&D activities remains low, confining it to the base of the GVCs. Consequently, there has been a continuous decline in China’s labor income share.

The results found in this paper hold significant policy implications for nations engaged in international trade, aiming to enhance the domestic labor market and share of labor income. For China, measures should focus on enhancing the quality and value-added of domestic service outsourcing activities. Additionally, considering the labor market’s role, the government should address unemployment and income inequality issues to mitigate the adverse effects of service outsourcing. Leveraging the positive impacts of offshore service outsourcing, China should enhance trade liberalization efforts and foster international cooperation and knowledge exchange in the realm of service outsourcing.

## 6. Conclusion

The expanding literature on GVCs and growing instances of service outsourcing, such as Chery, has reshaped the comprehension of the influence of value-added from production factors on labor income share. Nonetheless, the role of outsourcing in this context is evolving. This paper explores a novel perspective on the GVCs and the impact of service outsourcing on the labor income share. Additionally, this paper investigates whether service outsourcing can mitigate or potentially reverse the decline in global labor income share.

In the majority of the nations, both onshore and offshore service outsourcing increases the labor income share. Moreover, this paper proves that service outsourcing can increase a country’s labor income share by affecting the labor market. Heterogeneous effects show outsourcing can boost labor income share, especially for less developed countries and low-tech industries. Particularly, after the financial crisis in 2008, offshore outsourcing’s impact on labor income share magnified. However, in China, service outsourcing diminishes labor income share. Future research should explore further in the Chinese context. This paper contributes to the study of the influencing factors of labor income share and GVCs decomposing.

The conclusions drawn from this study echo the managerial insights presented in the preceding section, such as the need for supportive government measures and the importance of enterprise expansion in service outsourcing. These insights are crucial for shaping policies and business strategies that can optimize the benefits of service outsourcing while mitigating potential adverse effects on labor income share. In essence, our findings underscore that both onshore and offshore service outsourcing have the potential to elevate labor income share in most nations, with notable exceptions like China where the impact is inverse.

Future research directions could benefit from further disentangling the decomposition of value added within GVCs, examining how the benefits and distribution of labor income are influenced by the evolving structures of international trade and investment. It is also essential to investigate how technological advancements and digitalization within GVCs reshape the labor market across different economies. Moreover, a deeper exploration into the role of service outsourcing in facilitating the upgrading of domestic industries within GVCs could provide insights into promoting sustainable economic growth and equitable income distribution.

## Supporting information

S1 Appendix (DOCX)

S2 Appendix (DOCX)

S3 Appendix (XLS)
