# Supplementary material for: The effect of service outsourcing on labor income share: Measuring labor income share from the global value chains perspective
Source: PLoS One. 2024 Sep 11;19(9):e0309656. doi: 10.1371/journal.pone.0309656 (PMC11389904; doi:10.1371/journal.pone.0309656)
Supplement: S1 Appendix — (DOCX) [file pone.0309656.s001.docx]

| **List of acronyms and abbreviations** | | |
| --- | --- | --- |
| **Variable** | **Variable (full name)** | **Description** |
| OECD | Organization for Economic Co-operation and Development | An international Organization. |
| FDI | Foreign direct investment | is defined by the International Monetary Fund as A “cross-border investment” in which an investor that is “resident in one economy [has] control or a significant degree of influence on the management of an enterprise that is resident in another economy”. |
| GDP | Gross Domestic Product | The total value of goods produced and services provided in a country during one year. |
| GVCs | global value chains | GVCs is defined by OECD as the different stages of the production process are located across different countries. |
| OECD- ICIO | OECD Inter-Country Input-Output (ICIO) | Inter-Country Input-Output (ICIO) has 45 unique industries based on ISIC Revision 4. The tables are provided for 76 countries (and Rest of the World). Data can be downloaded in http://oe.cd/icio |
| WIOD | World Input-Output Database | World Input-Output Tables and underlying data, covering 43 countries, and a model for the rest of the world for the period 2000-2014 (WIOD 2016 Release). Data for 56 sectors are classified according to the International Standard Industrial Classification revision 4 (ISIC Rev. 4). Data can be downloaded in www.wiod.org. |
| ICIO | Inter-Country Input-Output | Inter-Country Input-Output table. Data can be downloaded in http://oe.cd/icio |
| WTO | World Trade Organization | The World Trade Organization deals with the global rules of trade between nations. |
| WITS | World Integrated Trade Solution | World Integrated Trade Solution is a software program provided by the World Bank. It is also a gateway to merchandise trade and protection statistics through the databases listed in https://wits.worldbank.org/about_wits.html |
| WDI | World Development Indicators | World Development Indicators is the primary World Bank collection of development indicators, compiled from officially recognized international sources. Data can be downloaded in https://databank.worldbank.org |
| KWW | Koopman, Wang, and Wei | Decomposition measure introduced by Koopman, Wang, and Wei (2014) |
| WWZ | Wang, Wei, and Zhu | Decomposition measure introduced by Wang, Wei, and Zhu (2013) |
| OLS | Ordinary least squares | Ordinary Least Squares is the most common estimation method for linear models. |
| 2SLS | Two-Stage Least Squares | Two-Stage Least Squares is the extension of the OLS method. |
| DVA | domestic value added | domestic value added decomposed from gross exports. |
| LVA | Labor value added | labor value added decomposed from gross exports. |
| CVA | Capital value added | Capital value added decomposed from gross exports. |
| LVAR | Labor value added ratio | labor income share decomposed from gross exports. |
| CVAR | Capital value added ratio | Capital income share decomposed from gross exports. |
| LPLV | Labor production length of value added | Labor production length of value-added measures the average number of times that value-added associated with labor factor in a country sector is counted as gross output along a production chain, until it is embodied in final products. |
