# Supplementary material for: The effect of service outsourcing on labor income share: Measuring labor income share from the global value chains perspective
Source: PLoS One. 2024 Sep 11;19(9):e0309656. doi: 10.1371/journal.pone.0309656 (PMC11389904; doi:10.1371/journal.pone.0309656)
Supplement: S2 Appendix — (DOCX) [file pone.0309656.s002.docx]

S2 Appendix:

## **Mechanisms**

The results of second stage of mechanism are reported in S2 Table 1.

**S2 Table 1. Mechanism (the second stage)**

| Dependent variable: | $LVAR$ | | | | $LPLV$ | | | |
| --- | --- | --- | --- | --- | --- | --- | --- | --- |
|  | （1） | （2） | （3） | （4） | （5） | （6） | （7） | （8） |
| lnEMP | 0.0389*** |  |  |  | 1.5459*** |  |  |  |
|  | (0.0025) |  |  |  | (0.4268) |  |  |  |
| lnEMPE |  | 0.0361*** |  |  |  | 1.4819*** |  |  |
|  |  | (0.0024) |  |  |  | (0.4089) |  |  |
| lnH_EMPE |  |  | 0.0348*** |  |  |  | 1.3777*** |  |
|  |  |  | (0.0024) |  |  |  | (0.3897) |  |
| lnINCOME |  |  |  | 0.0566*** |  |  |  | 0.9349*** |
|  |  |  |  | (0.0035) |  |  |  | (0.3101) |
| Controls | Yes | Yes | Yes | Yes | Yes | Yes | Yes | Yes |
| Fixed effects | Yes | Yes | Yes | Yes | Yes | Yes | Yes | Yes |
| R Square | 0.7231 | 0.7207 | 0.7197 | 0.7411 | 0.1347 | 0.1331 | 0.1297 | 0.1137 |
| Observations | 9994 | 9994 | 9994 | 9994 | 9993 | 9993 | 9993 | 9993 |

*Controls includes* all the above-mentioned control variables. *Fixed effects* include year fixed effects, country fixed effects, and industry fixed effects. Robust standard errors are in parentheses. ***, **, * stand for the significance level at 1%, 5% and 10%.

## **Heterogeneous effects**

***Time*** ***heterogeneity***. The benchmark results document a positive impact of service outsourcing on labor share income. However, studies in the literature also found some exogenous shocks that have an impact on manufacturing labor income and thus affect the benchmark results. The most likely exogenous shock to have such an impact on the time horizon of this paper is the global financial crisis in 2008. Therefore, the sample is divided into pre-2008 and post-2008, and the regression results are shown in S2 Table 2. Comparing column 1 and column 2 with column 3 and 4, the coefficients are consistent with the main regression results, the services outsourcing facilitates the movement of a country's labor share income. Besides, the forms of service outsourcing are turning into marked differences in their effects after the financial crisis. In other words, although the growth rate of production globalization declined with the financial crisis, service outsourcing can still contribute to the further strengthening labor share income.

**S2 Table 2. Time heterogeneity**

| Dependent variable: $LVAR$ | 2000-2007 | | 2008-2014 | |
| --- | --- | --- | --- | --- |
|  | （1） | （2） | （3） | （4） |
| $\begin{matrix} Offshore \end{matrix}$ | 0.6255*** |  | 0.7454*** |  |
|  | (0.1559) |  | (0.1328) |  |
| $\begin{matrix} Onshore \end{matrix}$ |  | 0.1571*** |  | 0.1348*** |
|  |  | (0.0219) |  | (0.0217) |
| $p$-value for  H_0_ : $\tilde{\beta}_{\left(1\right)}$= $\tilde{\beta}_{\left(2\right)}$ | 0.0900* | 0.0100 |  |  |
| $p$-value of  H_0_ : $\tilde{\beta}_{off}$= $\tilde{\beta}_{on}$= 0 | 0.3695 |  | 0.0011** |  |
| Controls | Yes | Yes | Yes | Yes |
| Fixed effects | Yes | Yes | Yes | Yes |
| R Square | 0.6923 | 0.6945 | 0.7378 | 0.7361 |
| Observations | 5148 | 5148 | 4846 | 4846 |

*Controls includes* all the above-mentioned control variables. *Fixed effects* include year fixed effects, country fixed effects, and industry fixed effects. $\tilde{\beta}_{\left(1\right)}$ refers to the coefficient of group 1, here means the group of 2000-2007. $p$-value are based on the F-test of the H_0_. Robust standard errors are in parentheses. ***, **, * stand for the significance level at 1%, 5% and 10%.

***Industrial heterogeneity****.*We then conduct separate regressions for different Industries. In this paper, manufacturing industries are classified into medium-high and low-technology manufacturing industries according to their technology levels. The results are reported in S2 Table 3.

The regression results show that service outsourcing in manufacturing industries of different technology levels all have a significantly positive effect on labor income share. In particular, the coefficients are significantly larger in the low-tech manufacturing sample, indicating that service outsourcing can more strongly promote labor in low-tech manufacturing industries to move up the value chain to higher value-added segments. Besides, the form of service outsourcing does not have a significant effect on its effect in medium and high technology industries.

**S2 Table 3. Industrial heterogeneity**

| Dependent variable: $LVAR$ | Medium & High Tech | | Low Tech | |
| --- | --- | --- | --- | --- |
|  | （1） | （2） | （3） | （4） |
| $\begin{matrix} Offshore \end{matrix}$ | 0.2700* |  | 0.7248*** |  |
|  | (0.1443) |  | (0.1205) |  |
| $\begin{matrix} Onshore \end{matrix}$ |  | 0.0528** |  | 0.2235*** |
|  |  | (0.0218) |  | (0.0215) |
| $p$-value for  H_0_ : $\tilde{\beta}_{\left(1\right)}$= $\tilde{\beta}_{\left(2\right)}$ | 0.0000*** | 0.0000*** |  |  |
| $p$-value of  H_0_ : $\tilde{\beta}_{off}$= $\tilde{\beta}_{on}$= 0 | 0.4114 |  | 0.0473** |  |
| Controls | Yes | Yes | Yes | Yes |
| Fixed effects | Yes | Yes | Yes | Yes |
| R Square | 0.7024 | 0.7025 | 0.7194 | 0.7216 |
| Observations | 4101 | 4101 | 5300 | 5300 |

*Controls* includes all the above-mentioned control variables. *Fixed effects* include year fixed effects, country fixed effects, and industry fixed effects. $\tilde{\beta}_{\left(1\right)}$ refers to the coefficient of group 1, here means the group of Medium & High Tech. $p$-value are based on the F-test of the H_0_. Robust standard errors are in parentheses. ***, **, * stand for the significance level at 1%, 5% and 10%.

***Service type heterogeneity****.*We also examined service type heterogeneity in S2 Table 4. The joint development of advanced manufacturing and modern services is manifested in the industrial source composition of investment goods, in which the share of broadly defined business services continues to rise. To explore the differences in the impact of different service activities on labor income share, this section divides service activities into business and non-business according to the OECD division criteria and conducts separate regressions, with the results in S2 Table 4. Clearly, the results do not differ significantly by distinguishing the types of services and the results of both groups indicate that the effect of offshoring is significantly higher than that of onshoring, and onshore service outsourcing of business services is significantly higher than non-business services.

**S2 Table 4. Service type heterogeneity**

| Dependent variable: $LVAR$ | Business Services | | Non-Business Services | |
| --- | --- | --- | --- | --- |
|  | （1） | （2） | （3） | （4） |
| $\begin{matrix} Offshore \end{matrix}$ | 0.5255*** |  | 0.5616*** |  |
|  | (0.1590) |  | (0.1034) |  |
| $\begin{matrix} Onshore \end{matrix}$ |  | 0.1670*** |  | 0.0733*** |
|  |  | (0.0199) |  | (0.0221) |
| $p$-value for  H_0_ : $\tilde{\beta}_{\left(1\right)}$= $\tilde{\beta}_{\left(2\right)}$ | 0.5928 | 0.0031** |  |  |
| $p$-value of  H_0_ : $\tilde{\beta}_{off}$= $\tilde{\beta}_{on}$= 0 | 0.0000*** |  | 0.0000*** |  |
| Controls | Yes | Yes | Yes | Yes |
| Fixed effects | Yes | Yes | Yes | Yes |
| R Square | 0.7021 | 0.7051 | 0.7044 | 0.7020 |
| Observations | 9994 | 9994 | 9994 | 9994 |

*Controls* includes all the above-mentioned control variables. *Fixed effects* include year fixed effects, country fixed effects, and industry fixed effects. $\tilde{\beta}_{\left(1\right)}$ refers to the coefficient of group 1, here means the group of Business Services. $p$-value are based on the F-test of the H_0_. Robust standard errors are in parentheses. ***, **, * stand for the significance level at 1%, 5% and 10%.
